# HYG-mol: An Interpretable Multimodal Hypergraph Framework for Molecular Property Prediction

**DOI:** 10.34133/csbj.0036

**Published:** 2026-04-09

**Authors:** Jiani Ma, Qi Yang, Lin Zhang, Hui Liu, Yuanting Zheng

**Affiliations:** ^1^ School of Information and Control Engineering, China University of Mining and Technology, Xuzhou 221116, P. R. China.; ^2^ National Key Laboratory of Agricultural Microbiology, College of Veterinary Medicine, Huazhong Agricultural University, 430070 Wuhan, Hubei, P. R. China.; ^3^ Faculty of Science, Melbourne Veterinary School, The University of Melbourne, Melbourne, Victoria, Australia.

## Abstract

Accurate molecular property prediction is a central task in drug discovery, yet existing methods often struggle to simultaneously capture higher-order molecular structures and chemically grounded semantic information. Graph neural networks are limited to pairwise atomic interactions, while Simplified Molecular Input Line Entry System-based language models lack explicit structural grounding, leading to incomplete structure–property representations. In this work, we propose HYG-mol, an interpretable molecular property prediction framework that integrates hypergraph-based structural modeling with multimodal chemical semantics. Molecules are represented as hypergraphs in which chemically meaningful substructures, such as functional groups and ring systems, are explicitly encoded as hyperedges, enabling direct modeling of higher-order structural dependencies. Chemical semantic information derived from a pretrained language model is fused with physicochemical descriptors at the atomic level. A hypergraph attention network is employed to capture cross-scale interactions and to identify substructures relevant to the prediction task. Extensive evaluations on MoleculeNet benchmark datasets demonstrate that HYG-mol consistently outperforms state-of-the-art baseline methods across both classification and regression tasks. Ablation and interpretability analyses further validate the effectiveness of the proposed representation and reveal strong correspondence between model-identified substructures and chemically meaningful motifs. Overall, HYG-mol provides a unified and interpretable framework for molecular property prediction by explicitly grounding chemical semantics in higher-order structural representations.

## Introduction

Predicting molecular properties from molecular structures is a fundamental task in drug discovery, and its accuracy directly impacts the efficiency and overall success of new drug development [1,2]. In recent years, rapid advances in artificial intelligence, particularly the deep integration of deep learning methods into molecular science, have established a new paradigm for molecular property prediction [3–5]. Despite notable progress, accurately characterizing molecular structures and effectively capturing their complex correlations with physicochemical and biological properties remains a central challenge for high-precision molecular property prediction.

Traditional molecular representations are dominated by manually designed features, such as fingerprint-based methods including Morgan fingerprints [6], Molecular Access System keys [7], and extended-connectivity fingerprints [8]. Although these methods have their own advantages in local topological encoding or computational efficiency, they all rely on manual feature engineering, fail to adaptively learn task-relevant molecular representations, and struggle to capture long-range structural interactions or distinguish complex molecules. The emergence of graph neural networks (GNNs) [9,10] has broken through the limitations of handcrafted features. By modeling molecules as graph structures of “atoms–chemical bonds”, GNNs explicitly model interatomic interactions through the message-passing mechanisms of models such as graph convolutional networks [11], graph attention networks [12], and graph isomorphism networks [13], achieving superior prediction performance than traditional methods. A fundamental limitation of most GNN-based approaches is that they are not explicitly designed to model multiatom, higher-order structures (e.g., functional groups and ring systems) as cohesive units, as message passing is primarily performed at the level of atomic pairs. However, many essential molecular properties, such as hydrophilicity and biological activity, are strongly associated with higher-order structures [14]. Even improved models like directional message-passing neural networks [15] and Attentive Fingerprint [16], which optimize message-passing methods, ultimately rely on the aggregation of atomic features to generate representations, lacking the ability to explicitly model the collective effects of high-order structures, which restricts the depth of structure–activity relationship learning. Parallel to graph-based approaches, transformer-based models operating on SMILES) sequences, such as MolBERT [17] and ChemBERTa [18], have attracted increasing attention. By leveraging large-scale self-supervised language modeling, these methods can learn rich contextual and semantic representations of molecules. However, representing molecules as one-dimensional sequences inevitably obscures explicit topological and spatial information [19]. As a result, the learned semantic representations cannot be reliably grounded in specific high-order structural units, such as functional groups or ring systems, limiting their ability to capture structure–property relationships that critically depend on precise molecular topology [20].

More importantly, the inability to explicitly chemical semantics in well-defined molecular structures remains a fundamental challenge for accurate molecular property prediction. To mitigate this issue, several studies have explored structural learning paradigms that attempt to implicitly incorporate semantic information. Self-supervised graph representation learning methods, such as MolCLR proposed by Wang et al. [21] and GROVER developed by Rong et al. [22], design contrastive and pretext tasks on molecular graphs to capture molecule-level contextual patterns, thereby enriching structural representations with implicit semantic information. In parallel, hierarchical graph models [23], including multilevel graph convolutional neural networks [24] and HiMol [25], aim to construct multiscale structural representations by aggregating atoms into higher-level substructures, through either predefined functional groups or implicitly learned hierarchies. Despite these advances, existing self-supervised and hierarchical graph models remain centered on atom-level representations, without explicitly defining high-order molecular substructures as representation units or modeling their semantic roles.

Existing molecular representation methods lack a unified framework that can explicitly model high-order molecular structures while integrating chemically meaningful semantics. To address these limitations, we propose HYG-mol, a molecular property prediction framework that explicitly integrates higher-order structural modeling with chemically grounded semantic representations. HYG-mol represents molecules as hypergraphs in which chemically meaningful substructures, such as functional groups and ring systems, are encoded as hyperedges, enabling direct modeling of interactions beyond pairwise atomic relationships. Contextual chemical representations derived from a pretrained language model are integrated with physicochemical features at the atomic level, allowing molecular topology to be coherently aligned with chemical semantics. Built upon this representation, a hypergraph attention network (HGAT) is developed to capture cross-scale dependencies and to identify substructures that are most relevant to molecular property prediction, thereby providing both improved predictive performance and interpretable structural insights.

## Methods

HYG-mol predicts molecular properties by representing molecules as hypergraphs and learning multilevel interactions through an HGAT. Hyperedges encode chemically meaningful substructures, including functional groups, rings, and inter-ring connections, each annotated with structured features capturing chemical type, size, aromaticity, electronic characteristics, and pharmacophore matching. Atoms serve as nodes with multimodal features that integrate ChemBERTa-derived semantic embeddings and RDKit-calculated physicochemical descriptors, which are standardized and fused. The resulting hypergraph is processed by a 3-layer hypergraph neural network with multihead attention. Node embeddings are aggregated via global pooling to produce a molecule-level representation, which is subsequently passed through linear layers for property prediction. The framework also supports interpretability by mapping learned representations back to molecular substructures.

### Molecular hypergraph construction and representation

#### Hyperedge representation

**Fig. 1.**
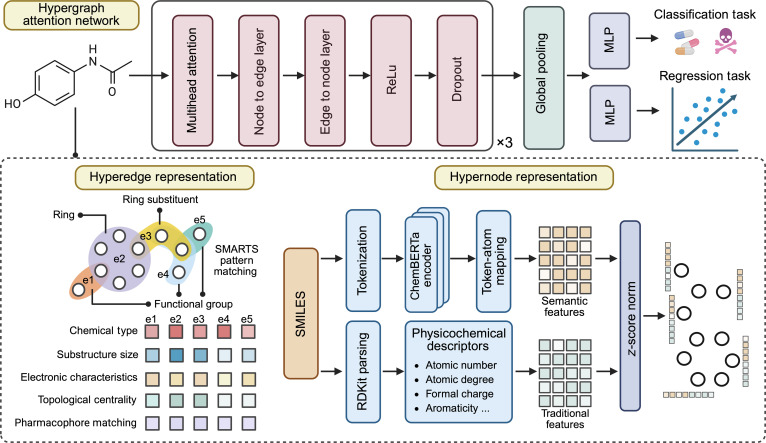
Overall architecture of the HYG-mol framework. Molecular structures are first converted into hypergraphs, where hyperedges represent chemically meaningful substructures (e.g., functional groups and rings) identified via SMILES Arbitrary Target Specification (SMARTS) patterns, with additional hyperedges for inter-ring connections and isolated atoms. Each hyperedge is associated with 5 features encoding chemical type, size, aromaticity, and electronic properties. Node features combine ChemBERTa-derived chemical semantic embeddings with physicochemical descriptors from RDKit, which are standardized and fused. The hypergraph is then processed by a 3-layer hypergraph neural network with multihead attention, followed by global pooling and a multilayer perceptron (MLP) for property prediction. The framework also allows interpretable analysis by mapping learned representations back to molecular substructures.

Traditional molecular graphs model atoms as nodes and chemical bonds as edges, capturing only pairwise interactions and failing to explicitly represent higher-order molecular substructures [26]. To overcome this limitation, HYG-mol employs a hypergraph-based molecular representation in which nodes correspond to atoms and hyperedges correspond to chemically meaningful substructures, providing a multilevel representation of molecular organization [27]. The overall framework of our model is illustrated in Fig. 1. Given a SMILES sequence, the molecule is first parsed into a molecular graph ***M*** using RDKit (version 2023.03.1) [28], and a hypergraph *H* = (*V*, *ε*) is subsequently constructed. Here, *V* = {*v*_1_,*v*_2_,...,*v_N_*} denotes the set of atomic nodes, and ε = {*e*_1_,*e*_2_,...,*e_M_*} denotes the set of hyperedges. Each hyperedge *e_k_* ⊆ *V* connects multiple atoms that collectively form a functional group, a ring system, or other biochemically relevant substructures, defined formally as *e_k_* = {*v_i_*|*v_i_* ∈ *S_k_*}, where *S_k_* is the set of atoms in the kth chemical substructure. Specifically, hyperedges are defined over the following chemically meaningful structural units:

• Functional groups: Functional groups are identified via RDKit-based SMILES Arbitrary Target Specification (SMARTS) substructure matching using a fixed and predefined library of 69 chemically meaningful SMARTS patterns. These patterns cover common functional groups, heterocycles, pharmacophoric motifs, and medicinal chemistry substructures (e.g., amines, esters, amides, aromatic and heteroaromatic rings, reactive alerts, and scaffold motifs). The same SMARTS library is applied uniformly across all datasets and tasks, without any dataset-specific tuning or adaptation. For a given molecule, all matched atom sets are deterministically encoded as hyperedges, allowing atoms to participate in multiple functional-group hyperedges when overlapping motifs are present.
• Ring systems: Aromatic, aliphatic, and polycyclic ring systems are encoded as hyperedges comprising all atoms within the ring, with additional annotations capturing ring size and aromaticity to preserve essential topological and electronic characteristics.
• Special structural motifs: Distinct hyperedges are constructed for special motifs such as metal coordination centers, spirocyclic systems, and bridged ring structures, allowing the model to explicitly represent nontrivial connectivity patterns that are difficult to capture with pairwise graphs.
• Ring connectivity: To characterize higher-order structural relationships, supplementary hyperedges are introduced to encode connections between adjacent rings as well as between rings and their substituents, thereby capturing intersubstructure dependencies.
• Isolated atoms: Atoms not assigned to any functional group or ring system are grouped with their directly bonded neighbors into hyperedges; atoms lacking bonded neighbors are represented as singleton hyperedges, ensuring complete atomic coverage.

While many hyperedges correspond to higher-order substructures such as functional groups and ring systems, pairwise atomic connections are naturally included as lower-order special cases, including chemically meaningful 2-atom substructures (e.g., carbon-heteroatom or carbon–halogen connections).

To fully leverage this detailed structural representation for molecular property prediction, each hyperedge is encoded as a 5d feature vector with its chemical and topological characteristics, including chemical type, substructure size, electronic characteristics, topological centrality, and pharmacophore matching. Specifically, chemical type encodes the substructure category, reflecting intrinsic properties such as polarity and stability. Substructure size, quantified by the normalized number of constituent atoms, serves as a proxy for steric effects and accessibility. Electronic characteristics are approximated by the weighted average of atomic electronegativities, capturing local electron density and reactivity. Topological centrality measures the connectivity of the hyperedge within the molecular hypergraph, highlighting core scaffolds that exert dominant influence on global molecular properties. Pharmacophore matching is represented as a binary indicator of whether the substructure conforms to established pharmacophore patterns, linking structural motifs to potential biological activity.

#### Multimodal hypernode representation

In HYG-mol, node features are constructed by integrating chemical semantic representations with physicochemical descriptors at the atomic level, forming a unified multimodal representation for each atom. To extract contextual chemical representation, SMILES sequences are first tokenized and encoded using a ChemBERTa model, yielding contextualized token-level representations ***z***_***t***_ for each token ***t***. As ChemBERTa generates token-level representations, a token-to-atom mapping is applied to project these embeddings onto atomic nodes. Atomic symbol tokens are directly assigned to their corresponding atoms, while subtokens prefixed with “##” are mapped to the same atoms as their parent tokens. When multiple tokens correspond to a single atom, their embeddings are aggregated using attention-weighted averaging to retain informative contributions:

$$h_{v}^{\text{BERT}}=\sum_{t\in T_{v}}\alpha_{t}z_{t}$$ (1)

where $h_{v}^{\text{BERT}}$ denotes the ChemBERTa-derived semantic feature of atom *v*, $T_{v}$ is the set of tokens mapped to v, and $\alpha_{t}$ is the normalized attention weight of token ***t***.

Due to the subword tokenization used by ChemBERTa, certain atomic symbols may be split into multiple context-dependent tokens (e.g., bracketed or composite atomic expressions), which prevents a direct one-to-one token-to-atom mapping in a small number of cases. In such rare cases, semantic features are inferred by averaging the semantic representations of their directly bonded neighbors:

$$h_{v}^{\text{BERT}}=\frac{1}{|N\left(v\right)|}\sum_{u\in N\left(v\right)}h_{u}^{\text{BERT}}$$ (2)

where |*N*(*v*)| denotes the set of neighboring items of *v*.

Through this mapping mechanism, contextual representations learned by ChemBERTa from large-scale chemical corpora are transferred from the SMILES token space to atom-level representations, thereby providing each atomic node with chemical context beyond local structural information.

In addition to semantic information, HYG-mol incorporates classical physicochemical descriptors to complement ChemBERTa-derived features. Specifically, the descriptors include basic attributes such as the normalized atomic number $Z_{i}/100$, degree $d_{i}/4$, and formal charge $q_{i}/8$; chemical properties such as aromaticity $I_{\text{aromatic}}\left(v_{i}\right)$ and the number of free electrons; and local environmental properties such as Gasteiger charge [29], ring membership, and the normalized number of bonded hydrogen atoms $H_{i}/4$. Each atom is further characterized by a normalized histogram of bond types among its incident bonds, which provides a compact summary of its local bonding configuration. To incorporate global molecular context, selected molecule-level properties, including molecular weight MW/500, octanol–water partition coefficient $\left(\log P\right)/10$,and topological polar surface area TPSA/100, are appended to each atomic descriptor. All aforementioned attributes are concatenated to form the traditional atomic feature vector $h_{v}^{\text{trad}}$. The normalization constants are selected based on typical upper bounds of the corresponding atomic attributes in organic molecules and are used to align feature scales for numerical stability, rather than to encode additional physical meaning.

The final node representation in HYG-mol is obtained by fusing the ChemBERTa-derived semantic feature vector $h_{v}^{\text{BERT}}$ with the traditional physicochemical descriptor vector $h_{v}^{\text{trad}}$. To mitigate scale disparities between feature modalities, *z*-score normalization is applied to each vector independently. The normalized features are then scaled by modality-specific weights and concatenated to produce the initial multimodal atomic representation:

$$h_{v}^{\left(0\right)}=\text{Concat}\left(w_{\text{BERT}}\cdot z\text{-score}\left(h_{v}^{\text{BERT}}\right),\;w_{\text{trad}}\cdot z\text{-score}\left(h_{v}^{\text{trad}}\right)\right)$$ (3)

where $h_{v}^{\left(0\right)}$ denotes the initial multimodal feature vector of node *v* and $w_{\text{BERT}}$ and $w_{\text{trad}}$ are hyperparameters controlling the relative contribution of semantic and physicochemical modalities.

### Hypergraph attention network

An HGAT is designed to model dependencies between atoms and chemically meaningful substructures within a molecular hypergraph using a multihead attention mechanism. It adopts a 2-stage propagation scheme, in which information is first aggregated from nodes to hyperedges and subsequently propagated back to nodes, enabling the integration of local and global structural information. The encoder consists of multiple stacked hypergraph convolutional layers, where symmetric attention weights are applied to node–hyperedge interactions to ensure consistent and interpretable message passing. Dropout is incorporated to mitigate overfitting. The detailed computation begins with the node-to-hyperedge aggregation stage.

In the node-to-hyperedge stage, the model first computes the raw attention score between each node *v* and hyperedge *e* for the *k*th attention head:

$$d_{\mathrm{ve}}^{k}=a_{v}^{k}\cdot \left(W_{v}^{k}h_{v}\right)+a_{e}^{k}\cdot \left(W_{e}^{k}f_{e}\right)$$ (4)

where $h_{v}$ and $f_{e}$ are the feature vectors of node *v* and hyperedge *e*, respectively; $W_{v}^{k}$ and $W_{e}^{k}$ are learnable linear transformations; and $a_{v}^{k}$ and $a_{e}^{k}$ are attention parameters. These raw scores are then passed through a LeakyReLU activation and normalized using a softmax function over all nodes within the same hyperedge:

$$\alpha_{\mathrm{ve}}^{k}={\text{softmax}}_{v\in e}\left(\text{LeakyReLU}\left(d_{\mathrm{ve}}^{k}\right)\right)$$ (5)

where $\alpha_{\mathrm{ve}}^{k}$ denotes the normalized attention coefficient, ensuring that the attention weights for nodes in each hyperedge sum to 1. Each hyperedge aggregates information from its constituent nodes to form a message vector:

$$m_{e\leftarrow V}^{k}=\sum_{v\in e}\alpha_{\mathrm{ve}}^{k}\left(W_{v}^{k}h_{v}\right)+W_{e}^{k}f_{e}$$ (6)

which combines the transformed node features with the hyperedge’s own features.

During the hyperedge-to-node stage, each node updates its representation by collecting messages from all incident hyperedges ℰ*_v_*, weighted by the corresponding attention scores:

$$m_{v\leftarrow E}^{k}=\sum_{e\in E_{v}}\alpha_{\mathrm{ve}}^{k}m_{e\leftarrow V}^{k}$$ (7)

where ℰ*_v_* denotes the set of hyperedge containing node *v*. The outputs from all *K* attention heads are concatenated, added to a learnable bias vector $b^{\left(l\right)}$, and passed through a nonlinear activation σ to yield the updated node features:

$$h_{v}^{\left(l+1\right)}=\sigma \left(\text{Conca}t_{k=1}^{K}\left(m_{v\leftarrow \varepsilon }^{k}\right)+b^{\left(l\right)}\right)$$ (8)

After *L* layers of propagation, a single molecular embedding is obtained through global average pooling over all node features:

$$h_{G}=\frac{1}{\left|V\right|}\sum_{v\in V}h_{v}^{\left(L\right)}$$ (9)

where $\left|V\right|$ is the total number of nodes in the hypergraph. This embedding $h_{G}$ captures the overall structural and chemical properties of the molecule and is finally passed into a 2-layer MLP to perform the property prediction task.

### Molecular interpretability analysis

To analyze how chemically meaningful substructures contribute to the predictions of HYG-mol, we derive interpretability signals directly from the attention mechanisms of HGAT. In HGAT, attention is defined on node–hyperedge pairs and quantifies the contribution of each atomic node to the representation of a connected chemical substructure during message passing. These node–hyperedge attention weights form the sole source of interpretability signals in our framework.

Based on the learned attention weights, interpretability is performed in a hierarchical manner. At the core, hyperedge-level importance scores quantify the contribution of each chemical substructure (e.g., functional groups or ring systems). These scores can be summarized at the molecule level to reflect the overall distribution of structural importance across the molecule and further propagated to the atomic level to obtain fine-grained atom-wise relevance patterns.

To reduce variability across attention heads and improve robustness, the attention coefficients are first averaged over all *K* heads at each propagation layer:

$${\bar{\alpha }}_{\mathrm{ve}}^{\left(l\right)}=\frac{1}{K}\sum_{k=1}^{K}\alpha_{\mathrm{ve}}^{k,\left(l\right)}$$ (10)

where ${\bar{\alpha }}_{\mathrm{ve}}^{\left(l\right)}$ is the averaged attention coefficient for the node–hyperedge pair (*v*, *e*) at layer *l*. Based on these aggregated attention weights, the contribution of each chemical substructure is quantified by computing the mean attention score of all nodes contained in the corresponding hyperedge. The hyperedge-level importance score is defined as

$$I\left(e\right)=\frac{1}{\left|e\right|}\sum_{v\in e}{\bar{\alpha }}_{\mathrm{ve}}^{\left(L\right)}$$ (11)

where $I\left(e\right)$ denotes the importance of hyperedge *e*, $\left|e\right|$ is the number of nodes in hyperedge *e*, and ${\bar{\alpha }}_{\mathrm{ve}}^{\left(L\right)}$ is the multihead averaged attention coefficient at the final propogation layer *L*.

For finer-grained interpretation, hyperedge-level importance scores are further propagated to the atomic level. The final importance of each atom is computed as the weighted sum of the scores of all hyperedges in which the atom participates. These atomic contributions can be visualized as a heatmap, highlighting key atoms and substructures that predominantly drive the model’s predictions.

## Results

### Experimental setting

Based on the MoleculeNet benchmark platform [30], 5 classification datasets, namely, Beta-Secretase 1 Inhibitors (BACE), Blood–Brain Barrier Penetration (BBBP), Toxicology in the 21st Century (Tox21), Side Effect Resource (SIDER), and Clinical Toxicity (ClinTox), and 5 regression datasets, namely, Estimated Solubility (ESOL), Free Energy of Solvation (FreeSolv), Lipophilicity, QM7, and QM8, were selected to comprehensively evaluate the performance of the proposed HYG-mol model across diverse molecular physicochemical properties and biological activities. These datasets vary substantially in size and task characteristics, providing a comprehensive benchmark for assessing model robustness under different data regimes. To rigorously assess the model’s generalization capability, all datasets were partitioned using the structure-based Bemis–Murcko scaffold splitting strategy [31], in which molecules are grouped according to their core scaffolds to minimize information leakage between structurally similar compounds across data splits. For each dataset, molecules were divided into training, validation, and test sets following an 80%–10%–10% split. For classification tasks, stratified splitting was applied to preserve the original class distribution across all subsets. This data partitioning scheme and experimental setup strictly follow the benchmark protocol adopted by HiMol. To ensure statistical robustness and account for training stochasticity, all experiments were independently repeated 5 times using 5 different random seeds. In this study, baseline methods covering different modeling strategies were selected for comparison. Graph-based molecular representation methods including GraphSAGE [32], AttributeMask [33], ContextPred [33], MoCL [34], GraphLoG [35], GraphCL [36], MolCLR [21], and MGSSL [37]. Hierarchical molecular modeling approaches are represented by HiMol [25]. Evaluation metrics follow the standard protocols of the MoleculeNet benchmarks and the corresponding baseline studies. For classification tasks, the area under the receiver operating characteristic curve (AUC-ROC) is used to evaluate a model’s ability to rank molecules according to their likelihood of exhibiting a target biological activity (e.g., active vs. inactive), independent of a fixed decision threshold. AUC-ROC measures the area under the curve defined by the true positive rate (TPR) and false positive rate (FPR),

$$\mathrm{TPR}=\mathrm{TP}/\left(\mathrm{TP}+\mathrm{FN}\right),\;\mathrm{FPR}=\mathrm{FP}/\left(\mathrm{FP}+\mathrm{TN}\right)$$ (12)

and can be interpreted as the probability that a randomly selected active compound is assigned a higher prediction score than a randomly selected inactive one. Higher AUC-ROC values indicate stronger discriminative and ranking capability, which is particularly suitable for class-imbalanced virtual screening scenarios.

For regression tasks, different evaluation metrics are adopted to reflect the physical nature of the target molecular properties. Root mean squared error (RMSE) is used for physical chemistry datasets (ESOL, FreeSolv, and Lipophilicity), where labels are subject to experimental or empirical noise and average prediction accuracy is of primary interest. Given ground-truth values $y_{i}$ and predictions ${\hat{y}}_{i}$ for *N* molecules, RMSE is defined as

$$\text{RMSE}=\sqrt{\frac{1}{N}\sum_{i=1}^{N}{\left({\hat{y}}_{i}-y_{i}\right)}^{2}}$$ (13)

Mean absolute error (MAE) is used for quantum mechanics datasets (QM7 and QM8), where targets are derived from high-precision quantum chemical calculations and larger prediction errors are particularly undesirable. MAE is defined as

$$\mathrm{MAE}=\frac{1}{N}\sum_{i=1}^{N}\left|{\hat{y}}_{i}-y_{i}\right|$$ (14)

For RMSE and MAE, lower values indicate better regression performance.

### Comparison experiments

**Fig. 2.**
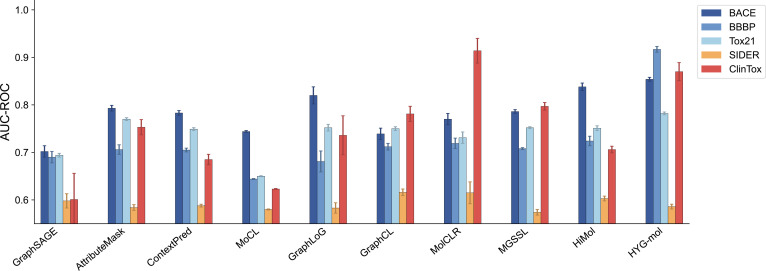
Performance comparison of HYG-mol and baseline models on classification tasks. Area under the receiver operating characteristic curve (AUC-ROC) performance of 13 models across 5 MoleculeNet classification datasets. Error bars indicate standard deviations.

To evaluate the predictive capability of HYG-mol across diverse molecular property prediction tasks, we conducted comprehensive comparison experiments against representative baseline models. Table 1 and Fig. 2 summarize the performance of HYG-mol on 5 classification benchmarks in comparison with multiple graph-based and self-supervised molecular representation methods. All results are reported as the mean performance over 5 independent runs with different random seeds, with standard deviations provided to reflect training stability.

**Table 1.** Performance comparison of HYG-mol and baseline models on classification datasets (%) averaged over 5 random seeds (mean ± SD). The best result is shown in bold. The second best results are underlined.

Model	BACE	BBBP	Tox21	SIDER	ClinTox
GraphSAGE	70.2 ± 1.2	69.0 ± 1.2	69.4 ± 0.4	59.8 ± 1.5	60.1 ± 5.5
AttributeMask	79.3 ± 0.6	70.6 ± 1.0	77.0 ± 0.3	58.4 ± 0.6	75.3 ± 1.6
ContextPred	78.3 ± 0.5	70.5 ± 0.4	74.9 ± 0.3	58.8 ± 0.3	68.5 ± 1.1
MoCL	74.4 ± 0.2	64.4 ± 0.1	65.0 ± 0.1	58.0 ± 0.1	62.3 ± 0.1
GraphLoG	82.0 ± 1.8	68.1 ± 2.2	75.2 ± 0.7	58.3 ± 1.1	73.6 ± 4.1
GraphCL	73.9 ± 1.2	71.2 ± 0.7	75.0 ± 0.4	61.6 ± 0.7	78.1 ± 1.6
MolCLR	77.0 ± 1.2	71.9 ± 1.1	73.1 ± 1.2	**61.5 ± 2.3**	**91.4 ± 2.6**
MGSSL	78.6 ± 0.4	70.8 ± 0.2	75.2 ± 0.2	57.4 ± 0.6	79.7 ± 0.8
HiMol	83.8 ± 0.8	72.4 ± 1.0	75.1 ± 0.5	60.3 ± 0.5	70.6 ± 0.7
HYG-mol	**85.4 ± 0.4**	**91.7 ± 0.6**	**78.2 ± 0.3**	58.6 ± 0.5	87.0 ± 1.9

BACE, Beta-Secretase 1 Inhibitors; BBBP, Blood–Brain Barrier Penetration; Tox21, Toxicology in the 21st Century; SIDER, Side Effect Resource; ClinTox, Clinical Toxicity.

HYG-mol achieves the best performance on most classification benchmarks, demonstrating clear advantages over existing molecular representation models. The most notable improvement is observed on the BBBP dataset, where HYG-mol achieves an AUC of 0.917, surpassing the second best model HiMol (0.724) by 26.7% and MolCLR (0.719) by 27.5%, highlighting the effectiveness of the proposed representation in capturing molecular patterns related to blood–brain barrier permeability. Consistent improvements are also observed on the BACE benchmark. HYG-mol achieves an AUC of 0.854, outperforming strong baselines such as HiMol (0.838) and GraphLoG (0.820).

For the Tox21 toxicity benchmark, HYG-mol achieves an average AUC-ROC of 0.782 across 12 prediction tasks. To provide a more detailed assessment of task-level performance, the AUC-ROC values for each individual Tox21 task are reported in Table S1 in the Supplementary Materials. As detailed in Table S1, the model shows particularly strong performance on several nuclear receptor-related assays, including NR-AR-LBD (0.897), SR-MMP (0.842), SR-ATAD5 (0.834), and NR-AR (0.825). These results indicate that the proposed hypergraph representation effectively captures structural patterns associated with receptor binding and cellular stress responses. In contrast, relatively lower performance is observed on more challenging pathways such as SR-HSE and NR-ER, which involve more complex regulatory mechanisms.

The results on ClinTox and SIDER further illustrate the robustness of the proposed framework under challenging prediction settings. ClinTox is characterized by severe class imbalance and limited training samples, yet HYG-mol still achieves strong predictive performance, suggesting that the learned representations remain effective even in low-data scenarios. For the SIDER benchmark, prediction performance remains relatively modest across most methods, reflecting the inherent difficulty of adverse drug reaction prediction. To provide additional insight into task-level variability, we report the AUC-ROC values for the 27 individual SIDER endpoints in Table S2 (Supplementary Materials). As shown in Table S2, predictive performance varies across adverse reaction categories, with relatively higher scores observed for tasks such as nervous system disorders (0.7092), renal and urinary disorders (0.7035), and skin and subcutaneous tissue disorders (0.6944), while lower scores appear for more heterogeneous endpoints such as product issues and cardiac disorders. These variations are consistent with the known difficulty and heterogeneity of adverse drug reaction prediction tasks. In addition, HYG-mol demonstrates stable performance across multiple training runs, as indicated by the relatively small standard deviations reported in Table 1. This consistency suggests that the proposed hypergraph-based representation provides robust molecular features that are less sensitive to random initialization. Overall, these results demonstrate that HYG-mol achieves reliable and consistent performance across diverse classification benchmarks, highlighting the advantage of explicitly modeling higher-order chemical substructures and integrating multimodal molecular features.

HYG-mol was further evaluated on 5 regression benchmarks, including ESOL, FreeSolv, Lipophilicity, QM7, and QM8, and the results are summarized in Table 2. Following the evaluation protocols adopted in previous studies, RMSE is used as the evaluation metric for ESOL, FreeSolv, and Lipophilicity, while MAE is used for QM7 and QM8. Similar to the classification experiments, all results are reported as the mean performance across 5 independent runs with different random seeds, with standard deviations indicating training stability.

**Table 2.** Performance comparison of HYG-mol and baseline models on regression datasets averaged over 5 random seeds (mean ± SD). The best result is shown in bold. The second best results are underlined.

Model	ESOL	FreeSolv	Lipophilicity	QM7	QM8
Metric	RMSE	RMSE	RMSE	MAE	MAE
GraphSAGE	2.048 ± 0.045	5.742 ± 0.123	0.807 ± 0.017	99.7 ± 3.9	0.020 ± 0.0001
AttributeMask	1.335 ± 0.021	7.598 ± 0.059	0.791 ± 0.007	225.9 ± 2.6	0.020 ± 0.0001
ContextPred	1.371 ± 0.005	6.884 ± 0.009	0.792 ± 0.005	204.6 ± 2.0	0.020 ± 0.0002
MoCL	1.432 ± 0.001	3.104 ± 0.001	1.234 ± 0.001	188.9 ± 28.4	0.059 ± 0.0135
GraphLoG	1.414 ± 0.011	3.070 ± 0.079	0.770 ± 0.002	254.7 ± 17.6	0.019 ± 0.0003
GraphCL	1.358 ± 0.028	3.279 ± 0.143	0.793 ± 0.005	226.0 ± 6.1	**0.019 ± 0.0001**
MolCLR	1.305 ± 0.031	2.707 ± 0.165	0.727 ± 0.010	112.1 ± 2.1	0.023 ± 0.0009
MGSSL	1.313 ± 0.006	3.083 ± 0.166	0.786 ± 0.006	157.4 ± 7.0	0.021 ± 0.0001
HiMol	0.938 ± 0.051	2.993 ± 0.127	0.773 ± 0.011	**95.1 ± 0.6**	0.020 ± 0.0004
HYG-mol	**0.692 ± 0.026**	**1.586 ± 0.011**	**0.671 ± 0.018**	106.6 ± 4.2	0.02 ± 0.0002

RMSE, root mean squared error; MAE, mean absolute error

For physicochemical property prediction tasks, HYG-mol consistently delivers the strongest performance among all compared models. On the ESOL dataset, HYG-mol achieves the lowest RMSE of 0.692, clearly surpassing the second best baseline HiMol (0.938) and reducing prediction errors substantially compared with widely used graph representation models such as MolCLR (1.305) and GraphCL (1.358). A similar trend is observed on the FreeSolv dataset. HYG-mol achieves an RMSE of 1.586, which is markedly lower than those of most baseline methods. In particular, the prediction errors of earlier self-supervised approaches such as AttributeMask (7.598) and ContextPred (6.884) remain considerably higher, indicating that HYG-mol provides a much more accurate estimation of hydration free energies. For the Lipophilicity dataset, HYG-mol again achieves the best result with an RMSE of 0.671, outperforming strong baselines including MolCLR (0.727) and GraphLoG (0.770). Overall, these results demonstrate that the proposed hypergraph-based representation is particularly effective for capturing molecular patterns associated with physicochemical properties.

For quantum chemistry prediction tasks, HYG-mol also demonstrates competitive performance. As summarized in Table 2, the model achieves prediction accuracy comparable to those of existing graph-based molecular representation methods on both QM7 and QM8 benchmarks, indicating that the proposed hypergraph representation maintains stable predictive capability across different types of molecular property prediction tasks. Overall, these results indicate that HYG-mol performs particularly well on physicochemical property prediction benchmarks while maintaining competitive performance on quantum chemistry tasks. The relatively small standard deviations across most datasets further suggest stable predictive behavior across different training runs. These findings highlight the advantage of modeling higher-order chemical substructures through hypergraph representations, which enables the model to capture complex structure–property relationships in molecular systems.

### Ablation experiments

#### Ablation studies on atomic representation

**Fig. 3.**
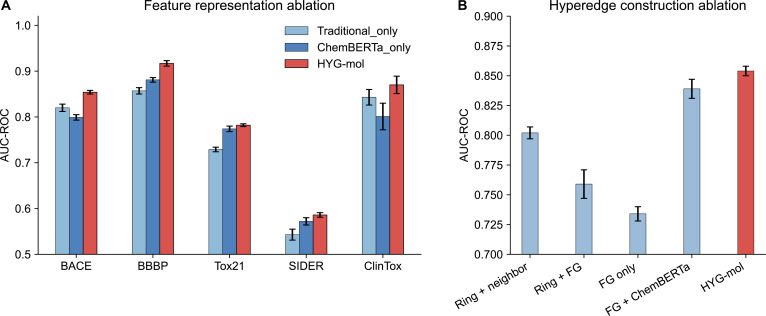
Results of ablation experiments. (A) Ablation analysis of feature engineering strategies. (B) Ablation analysis of hyperedge construction methods.

To investigate the contribution of different feature types in HYG-mol, we conducted ablation experiments by removing one feature source at a time. Specifically, Traditional_only denotes the variant that retains only the traditional physicochemical descriptors while removing ChemBERTa-derived semantic features, whereas ChemBERTa_only refers to the variant that keeps only the ChemBERTa features and excludes the traditional descriptors. The results are summarized in Table 3 and Fig. 3A.

**Table 3.** Performance comparison of feature engineering variants on classification datasets (%) averaged over 5 random seeds (mean ± SD). The best result is shown in bold.

Dataset	BACE	BBBP	Tox21	SIDER	ClinTox
Traditional_only	82.0 ± 0.8	85.7 ± 0.7	72.9 ± 0.5	54.3 ± 1.2	84.3 ± 1.7
ChemBERTa_only	79.9 ± 0.6	88.1 ± 0.5	77.4 ± 0.6	57.2 ± 0.8	80.1 ± 2.9
HYG-mol	**85.4 ± 0.4**	**91.7 ± 0.6**	**78.2 ± 0.3**	58.6 ± 0.5	87.0 ± 1.9

BACE, Beta-Secretase 1 Inhibitors; BBBP, Blood–Brain Barrier Penetration; Tox21, Toxicology in the 21st Century; SIDER, Side Effect Resource; ClinTox, Clinical Toxicity.

Removing either feature source leads to noticeable performance degradation across all datasets, indicating that both feature types contribute to the predictive capability of the model. Compared with the full HYG-mol model, removing ChemBERTa features (Traditional_only) results in AUC reductions of 3.4%, 6.0%, 5.3%, 4.3%, and 2.7% on BACE, BBBP, Tox21, SIDER, and ClinTox, respectively. Conversely, removing traditional physicochemical descriptors (ChemBERTa_only) leads to decreases of 5.5%, 3.6%, 0.8%, 1.4%, and 6.9% on the same datasets.

These results suggest that the 2 feature types contribute differently across tasks. The removal of ChemBERTa features causes larger performance degradation on 3 datasets (BBBP, Tox21, and SIDER), indicating that semantic molecular representations play a particularly important role in capturing complex chemical patterns. In contrast, removing traditional descriptors leads to larger drops on BACE and ClinTox, highlighting the importance of explicit physicochemical information for certain biological activity prediction tasks. Overall, the best performance is consistently achieved when both feature types are integrated, demonstrating that traditional descriptors and ChemBERTa-derived representations provide complementary molecular information that together enable more expressive molecular representations.

#### Ablation study on hypergraph construction strategies

Figure 3B and Table 4 summarize the results of ablation experiments designed to evaluate the influence of hypergraph construction strategies and hyperedge definitions on model performance. Four hypergraph construction variants were first assessed on the BACE dataset. Method 1, which integrates ring structures with atomic neighborhood information, achieved an AUC of 0.802, indicating that incorporating local atomic connectivity together with cyclic topology effectively captures fundamental molecular structural patterns. Method 2, combining ring structures with functional-group information, obtained a lower AUC of 0.759. When the hypergraph was constructed solely based on functional groups (method 3), performance further decreased to 0.734, suggesting that functional groups alone are insufficient to fully characterize the structural determinants associated with BACE inhibitory activity.

**Table 4.** Ablation experiments on hyperedge construction strategies for the BACE dataset (%) averaged over 5 random seeds (mean ± SD). The best result is shown in bold.

Hyperedge construction method	Ring structure	Atomic neighborhood	Functional group	ChemBERTa feature enhancement	AUC-ROC
1	√	√			80.2 ± 0.5
2	√		√		75.9 ± 1.2
3			√		73.4 ± 0.6
4			**√**	**√**	83.9 ± 0.8
HYG-mol	√	√	√	√	**85.4 ± 0.4**

BACE, Beta-Secretase 1 Inhibitors; AUC-ROC, area under the receiver operating characteristic curve

To investigate the contribution of semantic molecular representations, ChemBERTa-derived features were incorporated into method 3 (method 4). This enhancement substantially improved the AUC to 0.839, representing an absolute gain of 10.5 percentage points compared with that of method 3. This result highlights a clear complementary effect between structural hypergraph representations and contextual semantic features extracted from pretrained molecular language models. Finally, the complete HYG-mol framework, which jointly integrates optimized hyperedge construction (ring structures, atomic neighborhoods, and functional groups), multimodal node features, and the attention mechanism, achieved the best performance with an AUC of 0.854. This result surpasses method 4 by 1.5 percentage points, confirming that combining richer structural relations with enhanced atomic representations leads to further performance improvements.

### Outlier analysis

To further investigate the limitations of the proposed model, we conducted an outlier analysis on the regression tasks. Specifically, molecules with the largest prediction deviations were examined, and the top 20 samples ranked by absolute prediction error were identified. The corresponding molecular structures are listed in Table S3 in the Supplementary Materials. Inspection of these outlier molecules reveals several common structural characteristics. First, some molecules contain long aliphatic chains or alkyl-dominated scaffolds, such as CCC(C)(C)CO, CC(C)CCOC=O, and CCCC(=O)OC. These structures are relatively underrepresented in the training datasets and therefore lie outside the dominant chemical distribution learned by the model. Second, several outliers correspond to highly fused polycyclic aromatic systems, such as c1ccc2c(c1)c3ccccc3c4ccccc24. The complex topology of these molecules introduces long-range structural dependencies that are difficult to fully capture using 2-dimensional (2D) molecular representations. Third, a subset of molecules exhibits heavy halogen substitutions, for example, c2(Cl)c(Cl)c(Cl)c1nccnc1c2(Cl) and Brc1ccc(I)cc1. Such substitutions introduce strong electronic effects and are relatively rare in the training data. In addition, several molecules contain large and complex scaffolds resembling natural product–like architectures, including steroid-like frameworks and multi-ring cage systems. Taken together, these observations indicate that the largest prediction errors tend to occur for molecules located in sparsely represented regions of chemical space or possessing unusually complex structural motifs. This suggests that the observed errors primarily arise from the limited representation of such structural patterns in the training datasets, rather than the intrinsic limitations of the proposed modeling framework.

### Interpretability analysis

**Fig. 4.**
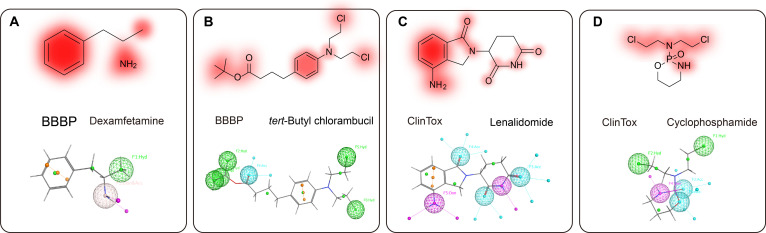
Interpretability analysis of the HYG-mol model This figure illustrates representative examples comparing the attention heatmaps generated by HYG-mol (top) with pharmacophore features identified by Molecular Operating Environment (MOE_ (bottom) for 4 molecules: (A) dexamfetamine, (B) *tert*-butyl chlorambucil, (C) lenalidomide, and (D) cyclophosphamide. In the attention maps, red regions indicate higher attention weights assigned to atoms or substructures, while the MOE pharmacophore annotations highlight hydrophobic regions (green spheres), hydrogen bond donors (magenta spheres), and hydrogen bond acceptors (cyan spheres). The comparison provides a qualitative view of how the structural regions emphasized by the model correspond to chemically relevant functional motifs.

To qualitatively examine whether the attention patterns learned by HYG-mol correspond to chemically meaningful substructures, we compared the attention distributions with pharmacophore features identified by Molecular Operating Environment (MOE) software version 2022.02 [38] for 4 representative molecules: dexamfetamine, *tert*-butyl chlorambucil, lenalidomide, and cyclophosphamide. Figure 4 illustrates the comparison between the attention heatmaps produced by HYG-mol and the corresponding MOE pharmacophore annotations for these molecules. For dexamfetamine, HYG-mol assigned the highest attention weight (0.900) to the primary amine group and the second highest weight (0.650) to the benzene ring scaffold. MOE pharmacophore analysis identified the hydrophobic region of the benzene ring (green Hyd sphere, F1) and the amine group exhibiting donor/hydrophobic properties (magenta Don sphere, F2), consistent with HYG-mol’s attention distribution. For *tert*-butyl chlorambucil, the model highlighted the nitrogen mustard group with the highest weight (0.900) and the *tert*-butyl ester protecting group with a moderate weight (0.450). Correspondingly, MOE detected hydrophobic features at F1 and F2 (*tert*-butyl group) and polar donor/acceptor features near the nitrogen mustard group (F3 and F4), with additional mixed polar features adjacent to the chlorine atom. For lenalidomide, the top 3 attention weights corresponded to the amide ring amino group (0.900), glutarimide ring carbonyl 1 (0.870), and carbonyl 2 (0.850). These assignments aligned with the “3-point anchoring” model of lenalidomide–cereblon binding reported in prior studies [39,40]. For cyclophosphamide, high attention weights were assigned to the 2 chlorine atoms of the nitrogen mustard group (0.850 and 0.830) and to the phosphoryl P=O group (0.600). MOE identified a clear separation between the hydrophobic aliphatic chain and the polar oxygen-containing region, with the chlorine atoms located at the highly reactive center bridging these regions. Overall, the attention maps learned by HYG-mol show qualitative correspondence with several pharmacophore features identified by MOE, suggesting that the model captures chemically meaningful structural motifs.

## Discussion

HYG-mol introduces a novel approach for molecular property prediction by representing molecules as hypergraphs, where chemically meaningful substructures such as functional groups and ring systems are explicitly encoded as hyperedges. Compared with conventional molecular graphs that model interactions primarily through pairwise atom–bond relationships, this formulation allows the model to directly represent higher-order structural motifs as unified structural units. Such motifs often play a critical role in determining physicochemical properties and biological activities. By explicitly modeling these multiatom substructures, the hypergraph representation enables the model to capture cooperative effects and structural dependencies that are difficult to express using standard graph-based message passing. The integration of ChemBERTa-derived semantic embeddings with classical physicochemical descriptors further enriches the node representations, allowing the model to combine contextual chemical knowledge with local structural information. Together, the hypergraph-based structural representation and multimodal node features provide a more expressive molecular representation, which is particularly beneficial for tasks where molecular properties are strongly influenced by functional groups, ring systems, and interactions between substructures.

Benchmark evaluations demonstrate that HYG-mol consistently outperforms state-of-the-art methods in both classification and regression tasks, particularly in scenarios requiring recognition of rare functional groups or long-range structural dependencies. Its hypergraph construction, combined with attention-guided message passing, allows direct modeling of key functional motifs and their contextual interactions, effectively bridging the gap between chemical knowledge and predictive modeling. Beyond predictive accuracy, HYG-mol’s design inherently supports interpretability. By aggregating multihead attention weights, the model quantifies the contribution of hyperedges to molecular properties and maps them to the atomic level. MOE pharmacophore analysis confirms that high-attention substructures correspond to chemically and biologically relevant motifs, offering a transparent and mechanistically grounded understanding of the model’s predictions. This feature enables data-driven guidance for structure–activity relationship studies, supporting experimental prioritization and rational molecular design.

Nevertheless, several limitations remain. First, HYG-mol currently relies solely on 2D molecular topology, which limits its ability to capture stereochemistry, conformational flexibility, and noncovalent interactions that are critical for binding affinity, enzyme specificity, and reaction mechanisms. In future work, this limitation could be addressed by combining hypergraph-based modeling for high-order 2D chemical topology with geometric or equivariant GNNs for explicit 3-dimensional structural information, allowing each representation to model the type of information that it is best suited for. Second, hyperedge identification depends on predefined SMARTS patterns, which may lead to incomplete coverage or bias when dealing with complex, macrocyclic, or organometallic compounds. Third, ChemBERTa-derived embeddings are constrained by the diversity and scope of their training corpus, potentially limiting generalization to novel chemical spaces. At a broader modeling level, although HYG-mol explicitly encodes higher-order chemical substructures through a unified hypergraph representation, it does not yet incorporate external chemical knowledge or adaptive hierarchical reasoning mechanisms, as explored in recent knowledge-guided and multiresolution molecular models [41,42]. As a result, the current framework may be less flexible in capturing context-dependent chemical semantics or dynamically adjusting representation granularity across different tasks. Fourth, although attention mechanisms in HYG-mol provide structure-aligned relevance signals over chemically meaningful hyperedges, recent studies have highlighted that attention weights alone may not constitute faithful or stable explanations of model behavior. In particular, attention-based interpretations may exhibit instability across training runs and may not fully reflect the causal contribution of structural components to predictions. Inspired by these findings, future work will extend the interpretability of HYG-mol by incorporating hyperedge-level perturbation and ablation analyses to explicitly evaluate the causal impact of functional substructures. In addition, gradient-based attribution methods, such as integrated gradients or gradient × input applied at the hyperedge level, will be explored as complementary explanation strategies. These extensions aim to move beyond attention visualization toward more robust and validated structure–property explanations.

Overall, we envision HYG-mol as a promising computational tool for informed molecular design and drug discovery, providing both predictive power and mechanistic insights that can guide experimental exploration and accelerate the development of novel molecules.

## Data Availability

The data used to support the findings of this study are available from the corresponding authors upon reasonable request. The molecular property data were derived from the MoleculeNet benchmark dataset (https://moleculenet.org/), which is publicly accessible. The code for the HYG-mol framework and related experimental scripts are deposited in GitHub (https://github.com/sutera777/HYG-mol).
