# Supplementary material for: HYG-mol: An Interpretable Multimodal Hypergraph Framework for Molecular Property Prediction
Source: Comput Struct Biotechnol J. 2026 Apr 9;35(1):0036. doi: 10.34133/csbj.0036 (PMC13062488; doi:10.34133/csbj.0036)
Supplement: Supplementary 1 — Tables S1 to S3 [file csbj.0036.f1.docx]

## **Supplementary Material**

## **HYG-mol: An Interpretable Multimodal Hypergraph Framework for Molecular Property Prediction**

Jiani Ma,^1^ Qi Yang,^1^ Lin Zhang,^1^ Hui Liu,^1, *^ and Yuanting Zheng^2, 3*^

^1^School of Information and Control Engineering, China University of Mining and Technology, Xuzhou, 221116, China

^2^ National Key Laboratory of Agricultural Microbiology, College of Veterinary Medicine, Huazhong Agricultural University, 430070, Wuhan, Hubei, P. R. China

^3^ Faculty of Science, Melbourne Veterinary School, The University of Melbourne, Parkville, Victoria, Australia

^*^ Correspondence: Hui Liu [hui.liu@cumt.edu.cn](mailto:hui.liu@cumt.edu.cn) , Yuanting Zheng yuanting.zheng1@unimelb.edu.au

**Table S1. ROC-AUC performance of HYG-mol on individual tasks of the Tox21 dataset.**

| **Task** | **Assay Description** | **ROC-AUC** |
| --- | --- | --- |
| nr-ar | Detects whether a compound activates the androgen receptor (AR) signaling pathway. | 0.8252 |
| nr-ar-lbd | Detects whether a compound binds to the ligand-binding domain (LBD) of the androgen receptor. | 0.8975 |
| nr-ahr | Detects whether a compound activates the aryl hydrocarbon receptor (AhR), which is involved in xenobiotic metabolism and detoxification pathways. | 0.8108 |
| nr-aromatase | Detects whether a compound inhibits aromatase, the enzyme responsible for converting androgens into estrogens. | 0.7522 |
| nr-er | Detects whether a compound activates the estrogen receptor (ER), a key regulator of hormonal signaling. | 0.6743 |
| nr-er-lbd | Detects whether a compound binds to the ligand-binding domain of the estrogen receptor. | 0.7773 |
| nr-ppar-gamma | Detects whether a compound activates peroxisome proliferator-activated receptor gamma (PPAR-γ), which regulates lipid metabolism and glucose homeostasis. | 0.7649 |
| sr-are | Detects activation of the antioxidant response element (ARE) pathway associated with oxidative stress response. | 0.7236 |
| sr-atad5 | Detects activation of ATAD5-mediated DNA damage response signaling. | 0.8336 |
| sr-hse | Detects activation of the heat shock response pathway mediated by heat shock elements (HSE). | 0.6670 |
| sr-mmp | Detects disruption of mitochondrial membrane potential (MMP), an indicator of mitochondrial toxicity. | 0.8425 |
| sr-p53 | Detects activation of the p53 signaling pathway, which regulates cellular stress responses and genomic stability. | 0.8160 |

**Table S2. ROC-AUC performance of HYG-mol on individual tasks of the SIDER dataset.**

| **Task** | **Assay Description** | **ROC-AUC** |
| --- | --- | --- |
| hepatobiliary disorders | Detects adverse effects related to the liver, gallbladder, and bile duct systems. | 0.6581 |
| metabolism and nutrition disorders | Detects metabolic imbalances and nutritional deficiencies caused by the compound. | 0.5849 |
| product issues | Detects issues related to the drug product's physical properties or packaging. | 0.3901 |
| eye disorders | Detects adverse effects on vision and the physiological structures of the eye. | 0.5800 |
| investigations | Detects abnormal findings from clinical tests and laboratory examinations. | 0.4965 |
| musculoskeletal and connective tissue disorders | Detects adverse effects on muscles, bones, joints, and connective tissues. | 0.5902 |
| gastrointestinal disorders | Detects adverse effects on the digestive system and gastrointestinal tract. | 0.6808 |
| social circumstances | Detects non-medical issues related to the social environment or lifestyle. | 0.5327 |
| immune system disorders | Detects hypersensitivity, allergies, and other immune-mediated adverse reactions. | 0.5489 |
| reproductive system and breast disorders | Detects adverse effects on reproductive organs and breast tissues. | 0.5918 |
| neoplasms benign, malignant and unspecified | Detects the potential for the compound to induce tumors, cysts, or polyps. | 0.5074 |
| general disorders and administration site conditions | Detects systemic symptoms and reactions at the site of drug delivery. | 0.5689 |
| endocrine disorders | Detects disturbances in hormonal regulation and endocrine gland functions. | 0.5942 |
| surgical and medical procedures | Detects complications arising from medical interventions or surgical procedures. | 0.5563 |
| vascular disorders | Detects adverse effects on blood vessels and circulatory regulation. | 0.4836 |
| blood and lymphatic system disorders | Detects adverse effects on blood components and the lymphatic system. | 0.6901 |
| skin and subcutaneous tissue disorders | Detects adverse reactions of the skin, hair, and subcutaneous tissues. | 0.6944 |
| congenital, familial and genetic disorders | Detects birth defects or hereditary-related adverse health conditions. | 0.5525 |
| infections and infestations | Detects increased susceptibility to bacterial, viral, or fungal infections. | 0.6432 |
| respiratory, thoracic and mediastinal disorders | Detects adverse effects on the lungs and the respiratory system. | 0.5983 |
| psychiatric disorders | Detects mental, emotional, and behavioral adverse effects. | 0.6166 |
| renal and urinary disorders | Detects adverse effects on kidney function and the urinary system. | 0.7035 |
| pregnancy, puerperium and perinatal conditions | Detects adverse effects occurring during pregnancy or the perinatal period. | 0.5833 |
| ear and labyrinth disorders | Detects adverse effects on hearing and balance (vestibular system). | 0.5041 |
| cardiac disorders | Detects adverse effects on heart rhythm, structure, and cardiac function. | 0.4605 |
| nervous system disorders | Detects adverse effects on the central and peripheral nervous systems. | 0.7092 |
| injury, poisoning and procedural complications | Detects adverse outcomes resulting from injuries, toxicity, or medical treatment. | 0.6254 |

**Table S3. Top 20 molecules with the largest prediction errors in regression tasks.**

| **Index** | **SMILES** |
| --- | --- |
| 1 | CCC(C)(C)CO |
| 2 | C1=Cc2cccc3cccc1c23 |
| 3 | CCOC(=O)c1ccccc1S(=O) |
| 4 | CCCC(=O)OC |
| 5 | CNC(=O)C(C)SCCSP(=O)(OC)(OC) |
| 6 | OC(C1=CC2C5C(C1C2=C(c3ccccc3)c4ccccn4)C(=O)NC5=O)(c6ccccc6)c7ccccn7 |
| 7 | OCC(O)C2OC1OC(OC1C2O)C(Cl)(Cl)Cl |
| 8 | c2(Cl)c(Cl)c(Cl)c1nccnc1c2(Cl) |
| 9 | Cc2ncc1nccnc1n2 |
| 10 | CC1CC(C)C(=O)C(C1)C(O)CC2CC(=O)NC(=O)C2 |
| 11 | COP(=S)(OC)Oc1cc(Cl)c(I)cc1Cl |
| 12 | CC(=O)C1CCC2C3CCC4=CC(=O)CCC4(C)C3CCC12C |
| 13 | CC1(C)C(C=C(Cl)Cl)C1C(=O)OCc2cccc(Oc3ccccc3)c2 |
| 14 | C1CCc2ccccc2C1 |
| 15 | CC(C)CCOC=O |
| 16 | c1ccc2c(c1)c3ccccc3c4ccccc24 |
| 17 | Cc1c(F)c(F)c(COC(=O)C2C(C=C(Cl)C(F)(F)F)C2(C)C)c(F)c1F |
| 18 | c1ccc2c(c1)c3cccc4ccc5cccc2c5c43 |
| 19 | Cc1ncc(N(=O)=O)n1CCO |
| 20 | Brc1ccc(I)cc1 |
